# Enrichment of Eucalyptus oil nanoemulsion by micellar nanotechnology: transdermal analgesic activity using hot plate test in rats’ assay

**DOI:** 10.1038/s41598-019-50134-y

**Published:** 2019-09-23

**Authors:** Zarith Asyikin Abdul Aziz, Hasmida Mohd Nasir, Akil Ahmad, Siti Hamidah Mohd Setapar, Hafandi Ahmad, Mohd Hezmee Mohd Noor, Mohd Rafatullah, Asma Khatoon, Mohd Adnan Kausar, Irfan Ahmad, Shahida Khan, Majed Al-Shaeri, Ghulam Md Ashraf

**Affiliations:** 1 Centre of Lipid Engineering and Applied Research (CLEAR), Ibnusina Institute for Scientific and Industrial Research, Universiti Teknologi Malaysia, 81310 UTM Johor Bahru, Johor Malaysia; 2 School of Chemical and Energy Engineering, Faculty of Engineering, Universiti Teknologi Malaysia, 81310 UTM Johor Bahru, Johor Malaysia; 3 SHE Empire Sdn Bhd, No 44, Jalan Pulai Ria 2, Bandar Baru Kangkar Pulai, 81300 Skudai, Johor Malaysia; 4 Department of Veterinary Preclinical Science, Faculty of Veterinary Medicine, Universiti Putra Malaysia, 43400 UPM Serdang, Selangor Darul Ehsan, Malaysia; 5 School of Industrial Technology, Universiti Sains Malaysia, Penang, 11800 Malaysia; 6 Department of Biochemistry, College of Medicine, University of Hail, Hail, Saudi Arabia; 7 Department of Clinical Laboratory Science, College of Applied Medical Sciences, King Khalid University, Abha, Saudi Arabia; 8 Research center for Advanced Material Sciences, King Khalid University Abha, Abha, Saudi Arabia; 9 Applied Nutrition Group, King Fahd Medical Research Center, King Abdulaziz University, Jeddah, Saudi Arabia; 10 Department of Medical Laboratory Technology, Faculty of Applied Medical Sciences, King Abdulaziz University, Jeddah, Saudi Arabia; 11 Department of Biological Sciences, Faculty of Science, King Abdulaziz University, Jeddah, Saudi Arabia; 12 King Fahd Medical Research Center, King Abdulaziz University, Jeddah, Saudi Arabia

**Keywords:** Natural product synthesis, Experimental models of disease

## Abstract

*Eucalyptus globulus* is an aromatic medicinal plant which known for its 1,8-cineole main pharmacological constituent exhibits as natural analgesic agent. *Eucalyptus globulus*-loaded micellar nanoparticle was developed via spontaneous emulsification technique and further evaluation for its analgesic efficacy study, *in vivo* analgesic activity assay in rats. The nanoemulsion system containing *Eucalyptus*-micelles was optimized at different surfactant types (Tween 40, 60 and 80) and concentrations (3.0, 6.0, 9.0, 12.0, 15.0, and 18.0 wt. %). These formulations were characterized by thermodynamically stability, viscosity, micelles particle size, pH, and morphology structure. The spontaneous emulsification technique offered a greener micelles formation in nanoemulsion system by slowly titrated of organic phase, containing *Eucalyptus globulus* (active compound), grape seed oil (carrier oil) and hydrophilic surfactant into aqueous phase, and continuously stirred for 30 min to form a homogeneity solution. The characterizations evaluation revealed an optimized formulation with Tween 40 surfactant type at 9.0 wt. % of surfactant concentration promoted the most thermodynamic stability, smaller micelles particle size (d = 17.13 ± 0.035 nm) formed with spherical shape morphological structure, and suitable in viscosity (≈2.3 cP) and pH value (6.57) for transdermal purpose. The *in vivo* analgesic activity assay of optimized emulsion showed that the transdermal administration of micellar nanoparticle of *Eucalyptus globulus* on fore and hind limb of rats, possessed the central and peripheral analgesic effects by prolonged the rats pain responses towards the heat stimulus after being put on top of hot plate (55 °C), with longest time responses, 40.75 s at 60 min after treatment administration. Thus, this study demonstrated that micellar nanoparticle of *Eucalyptus globulus* formed in nanoemulsion system could be promising as an efficient transdermal nanocarrier for the analgesic therapy alternative.

## Introduction

Essential oils are part of medicinal plant, which becomes important within product produced in agricultural industry. Its chemical constituents are among secondary plant metabolite with lipophilic and high volatility characteristics^1^. These plant origin products are frequently used as formulation additive in foods, drinks, perfumeries, and cosmetics product^2,3^. Analgesic properties that naturally present as among main constituent of essential oils have been revealed by several researchers^4–7^. Some of essential oils were demonstrated with successful analgesic activities, which possess to be high potential as alternative of non-steroidal anti-inflammatory drugs (NSAIDs)^8,9^. NSAIDs are the synthetic analgesic drugs which widely commercialized in pharmaceutical industries and clinically proven to be consumed as a pain reliever agent for many ailments. However, the major adverse effect of NSAIDs are reported such as cardiovascular diseases^10^, kidney failures^11^, stroke^12^, stomach ulcers^13^, and hypertension^14^.

*Eucalyptus* is an aromatic plant species from Myrtaceae family, extracted for its essential oil for various purposes. In Brazil, *Eucalyptus* essential oil has broadly used as traditional medicine for numerous of medical diseases. Currently in Malaysia, *Eucalyptus* tree with several species have been planted in Sarawak with the area of 28,000 hectares^15^. According to Committee of Herbal Medicine Products (HMPC), a committee of European Medicines Agency reported the traditionally application dosage of *Eucalyptus globulus* essential oil via transdermal administration at ranges between 1.7–4 g/100 L of water^16^. This traditional application of *Eucalyptus globulus* essential oil was demonstrated for bath additive in the treatment of muscle aches that attributable to analgesic pharmacological activity of *Eucalyptus* essential oil. Besides, the main constituent of *Eucalyptus globulus* essential oil known as 1,8-cineole has been widely discussed for its natural analgesic properties^6^.

Transdermal or topical administration of essential oils possesses a successful and innovative route in herbal drug delivery system and known to enhance drugs therapeutic efficacy with tremendous advantageous compared to orally administration^17^. Due to this innovative delivery, hepatic metabolism can be avoided, easier administration, and achieving patience convenient and compliance^18^. Transdermal applications of essential oils are proven to be safe, however the lipophilic and high volatility characteristics of essential oils limit their efficacies, encourage it to be applied in excessive way to achieve good therapeutics for some treatments. Moreover, due to high volatility characteristic, essential oils are easily decomposed under heat, light, oxygen, and humidity exposures and resulted into unstable bioactive component, lower efficacy and bioavailability^19^. Related to these matters, Darben *et al*.^20^ has reported a toxicity of topical *Eucalyptus* experienced by a 6-years old Caucasians girl, resulted with nausea, slurred speech, ataxia, and muscle weaknesses after application of 25 mL of *Eucalyptus globulus* on her skin^20^.

Micellar nanoparticle is a nanotechnology-based formulation, with high achievement of transdermal therapeutics and able to form very fine nanoparticle achieving 10 nm^21^. The formulation offers a robust and versatile delivery system in ability to incorporate a range of lipophilic constituents having numerous physicochemical properties, as a nano-vehicle to deliver the lipophilic constituent efficiently onto skin, while protected the constituents from easily decomposed due to biological interaction in human body^22^. This nanocarrier developed as one of the latest nanotechnology-based in transdermal delivery system, compared to the other conventional nanocarriers such as liposomes, niosomes, and ethosomes which struggled with some challenges such as lower bioactive encapsulation efficiency, expensive in production and larger particle size^22–25^. Micelles can be formed by several methods such as solvent evaporation, dialysis method, solid dispersion, and oil in water emulsion^26^.

Nanoemulsion is among types of O/W emulsion with a better bioavailability approach in drug delivery system attributable to the system capability in forming fine micelles particle size at lower surfactant concentration^27^. By comparing with other methods, micelles formation through O/W nanoemulsion system is broadly investigated and suitable for transdermal administration purpose^27^. This nanocarrier system offers a relative high kinetic stability even for several years, due to their very tiny droplets and the consequence of significance steric stabilization between droplets^1^. The enhancement of pharmacological activities among essential oils are reported to be successful by enriching these aromatic with micellar nanoparticle through nanoemulsion system which mainly focusing on the antibacterial potential^19,28^.

Meanwhile, developing nanoemulsions with any high energy devices can be avoided by using low-energy techniques, an approach to form micelles spontaneously via rely on interfacial phenomenon between oil and water phases that suitable for components with labile properties like essential oil. Low-energy methods are consisting of phase inversion temperature (PIT), phase inversion composition (PIC), and spontaneous emulsification methods. These methods are not widely applied but some studies reported regarding their advantageous to form very tiny micelles droplets easily compared to high-energy approaches^29,30^. Previous investigations have shown the ability of spontaneous emulsification technique to form smaller micelles particle size that was depending on various factors such as system compositions, surfactant solubility, interfacial tension and surfactant phase behaviour^29^.

Therefore, the main purpose of the this study was to optimize the nanoemulsion system’s thermodynamic stability, viscosity, micelles particle size, pH measurement and morphology characterizations, depending on manipulated surfactants type and concentration. An optimize formulation with successful characterizations was chosen to be further tested for its *in vivo* transdermal analgesic potential evaluation using hot plate test in rats. Hence, this research promotes an innovative approach for a further implementation of *Eucalyptus globulus* essential oil in various fields, as the scientific findings of this research successfully revealed an enhancement of *Eucalyptus globulus* essential oil pharmacological activity (analgesic) can be achieved by fabricated with micellar nanoparticle technology.

## Results

### Gas chromatography-mass spectrometry (GC-MS) Analysis of eucalyptus globulus essential oil

**Figure 1:**
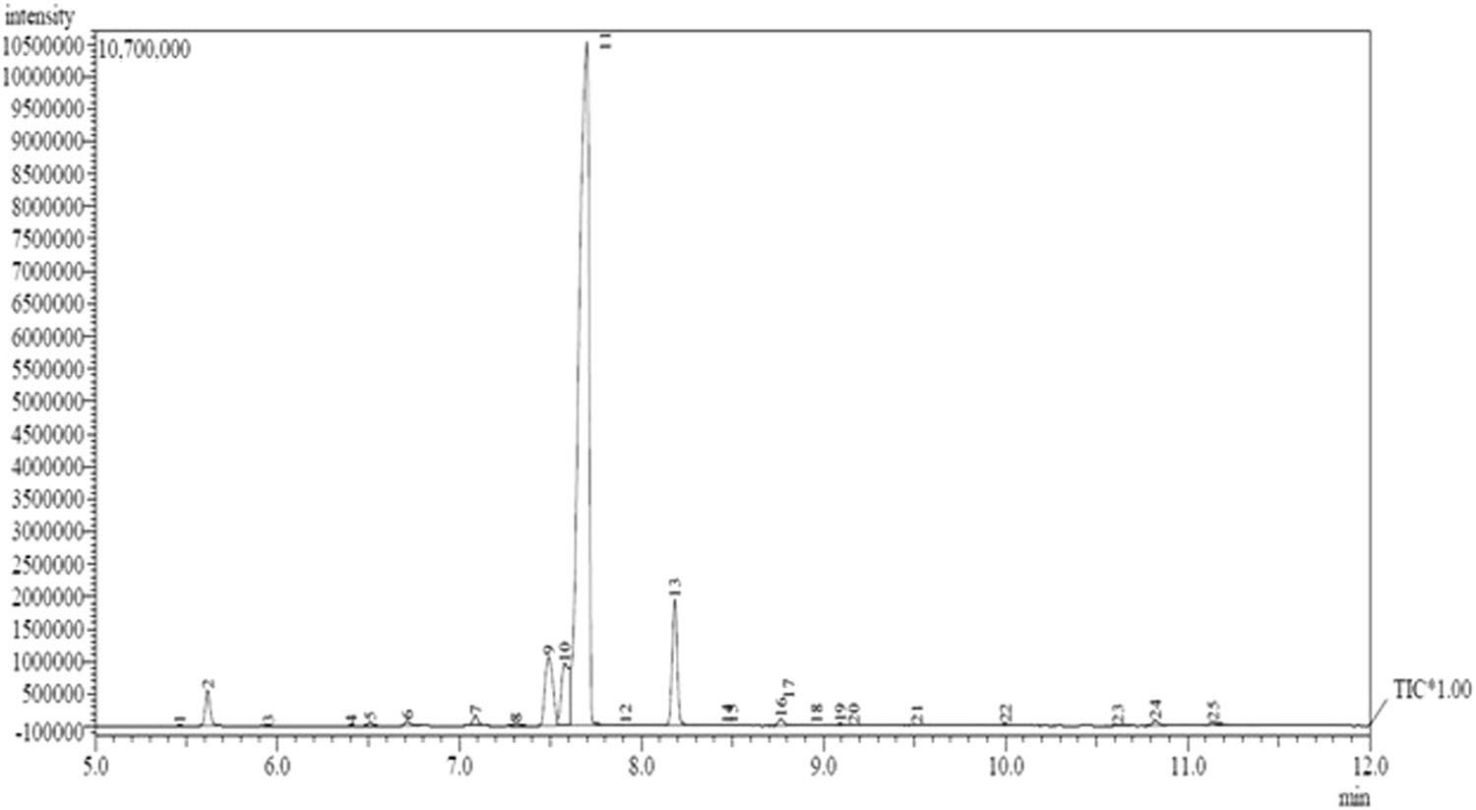
Gas chromatography-mass spectrometry (GC-MS) chromatograph of *Eucalyptus globulus* essential oil.

To determine the chemical constituents contain inside the *Eucalyptus globulus* essential oil, gas chromatography-mass spectrometry (GC-MS) analysis was accessed. The total *Eucalyptus globulus* essential oil ion chromatogram is illustrated as Fig. 1. The amount of chemical constituents contain from the aromatic oil were determined by peak area normalization method. Besides, the detail on *Eucalyptus* essential oil components presented in each peak are listed as Table 1.

**Table 1:** Chemical constituents of *Eucalyptus globulus* essential oil.

Peak No.	Retention Time (min)	Molecular Formula	Compound	Relative Content (%)
1	5.462	C_10_H_16_	α-Thujene	0.01
2	5.617	C_10_H_16_	α-Pinene	1.89
3	5.952	C_10_H_16_	Camphene	0.01
4	6.406	C_10_H_16_	Β-Phellandrene	0.01
5	6.510	C_10_H_16_	Β-Pinene	0.21
6	6.713	C_10_H_16_	Β-Myrcene	0.32
7	7.088	C_10_H_16_	α-Phellandrene	0.60
8	7.311	C_10_H_16_	Terpinolen	0.17
9	7.490	C_10_H_14_	Benzene	6.16
10	7.577	C_10_H_16_	D-Limonene	5.89
11	7.699	C_10_H_18_O	1,8-Cineole	75.96
12	7.913	C_10_H_16_	Β-Ocimene	0.06
13	8.183	C_10_H_16_	Gamma-Terpinene	7.04
14	8.471	C_10_H_18_O_2_	2-Furanmethanol	0.06
15	8.498	C_9_H_10_O_3_	3-Methyl-4-cyclohexene	0.05
16	8.766	C_10_H_16_	Terpinolen	0.43
17	8.810	C_10_H_18_O_2_	2-Furanmethanol	0.03
18	8.962	C_10_H_12_	Styrene	0.03
19	9.094	C_10_H_18_O	Β-Linalool	0.13
20	9.167	C_10_H_20_O_2_	Butanoic acid	0.07
21	9.515	C_10_H_18_O	D-Fenchyl alcohol	0.08
22	9.998	C_10_H_16_O	Bicyclo[3,1,1]heptan-3-ol	0.13
23	10.617	C_10_H_18_O	α-Terpineol	0.05
24	10.823	C_10_H_18_O	Terpinen-4-ol	0.34
25	11.139	C_10_H_18_O	Terpinen-4-ol	0.32

Depicting to the chromatograph finding, it was shown that dominated chemical group of *Eucalyptus* essential oil was mainly from oxygenated monoterpenes, a monoterpenes group attached with oxygen atom and followed by monoterpenes (C_10_H_16_). Among these, the main oxygenated monoterpenes reported to be contained 1,8-cineole (75.96%), and terpinen-4-ol (0.34%), while gamma-terpinene (7.04%), benzene (6.16%), D-limonene (5.89%), and α-Pinene (1.89%) as main monoterpenes group.

**Figure 2:**
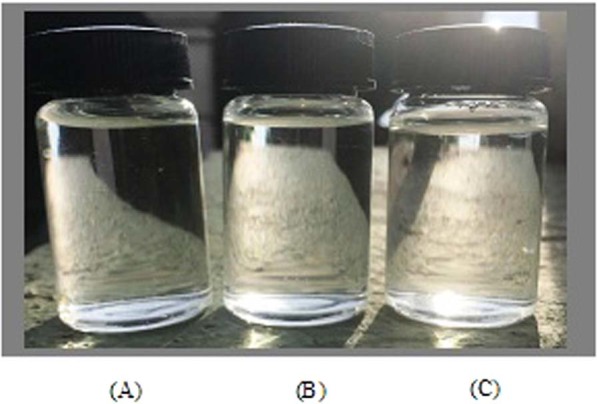
Transparent micellar formulation of *Eucalyptus globulus* essential oil in nanoemulsion system; (**A**) Tween 40, (**B**) Tween 60, and (**C**) Tween 80 of surfactants.

Therefore, the percentage of 1,8-cineole is significant with the values reported which almost similar with previous investigations. Song *et al*.^31^ demonstrated that the dominant constituents of *Eucalyptus globulus* leaves essential oil was 1,8-cineole with 72.71%. Besides, another study showed an essential oil of *Eucalyptus globulus* with 1,8-cineole as the highest plant constituent at 83.89%^32^. Regarding the natural analgesic pharmacological activity of *Eucalyptus globulus* essential oil related with the 1,8-cineole content, it have been initially scientifically proven by Juergens *et al*.^33^ and followed with several research studies^6,34^. Hence, the current results (Fig. 2 and Table 1) are significantly correlate with the previous literatures regarding 1,8-cineole content as major constituents of *Eucalyptus globulus* essential oil.

### Formulation of eucalyptus globulus-enriched micellar nanoparticle in nanoemulsion system

One of successful micellar formulation characteristic is ability of the nanoemulsion system to be formed as transparent solution, represent smaller micelles dispersed throughout aqueous phase. Spontaneous emulsification technique that implemented through this research study was successfully developed a transparent formulation of nanoemulsion system, contained dispersion of *Eucalyptus globulus* essential oil- loaded micellar nanoparticle through aqueous solution. This was achieved by good surfactant diffusion during titration process of organic to aqueous phase, which facilitates smaller *Eucalyptus-*micelles particle size and transparent formulation formed. Figure 2 shows the transparent nanoemulsion system formulated from the three surfactants type used (Tween 40, 60, and 80) at concentration of 12.0 wt. %. The three surfactants; Tween 40, 60 and 80 are among suitable surfactant to be used to form micelles in O/W nanoemulsion and preferable for transdermal application due to their non-toxic and non-irritant properties.

**Figure 3:**
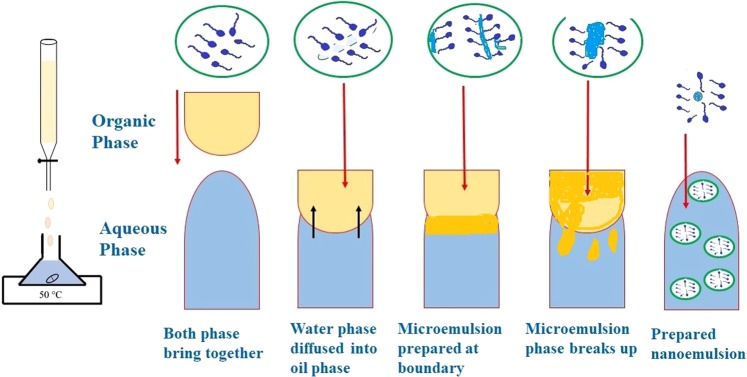
Schematic diagram of mechanism of micelles formation through nanoemulsion system using spontaneous emulsification technique.

This transparency illustration represent the smaller micelles dispersed through nanoemulsion system. Saberi *et al*.^29^ stated that the fine micelles particle size formed is often do not scatter light strongly, attributable to its smaller size than light wavelength (d « λ) and resulted to develop a transparent solution. Besides, this fact is supported by other researchers through development of carvacrol and orange essential oils- loaded micellar nanoparticle in nanoemulsion system^35,36^. Hence, Fig. 3 shows the illustration of potential mechanism for formation of micelles in nanoemulsion system during titration process of organic to aqueous phase via spontaneous emulsification technique.

Besides, by comparing with other emulsification technique, another transparent micellar formulation dispersed with smaller *Eucalyptus-*micelles particle size was also successfully developed using high-energy emulsification technique. Sugumar *et al*.^37^ formulated micellar nanoparticles of *Eucalyptus* oil in nanoemulsion using ultrasonication method to evaluate its antibacterial properties against *Staphylococcus aureus* and wound healing activity in Wistar rats. The result revealed an enhancement of antibacterial activity of *Eucalyptus* oil after fabricated with micellar nanoparticles. Even though smaller *Eucalyptus*-micelles can be formed using high-energy technique, researchers are mostly preferred to choose low-energy method to form micellar nanoparticles-enriched essential oil attributable to labile properties of essential oil. Therefore, this present study is relevant to be conducted by successfully developed transparent and smaller Eucalyptus-micellar nanoparticles formulation via simple stirring (spontaneous emulsification) method.

### Preliminary experiment

**Figure 4:**
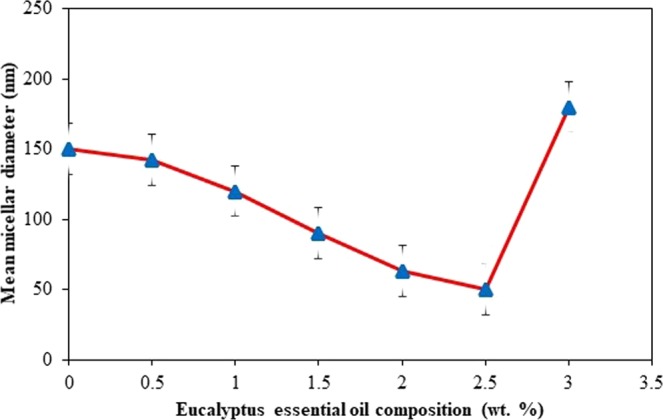
Effect of oil composition (wt. %) on micelles particle size (experimental conditions: 100 mL solution; 3.0 wt. % of oil composition, 3.0 wt. % of Tween 40 surfactant concentration, at 25 °C temperature and 1000 rpm stirring speed).

**Figure 5:**
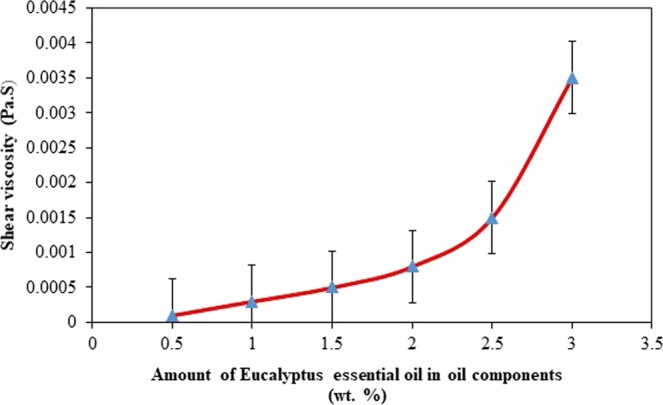
Shear viscosity related to *Eucalyptus* essential oil composition in oil phase (experimental conditions: 100 mL solution; 3.0 wt. % of oil composition, 3.0 wt. % of Tween 40 surfactant concentration, at 25 °C temperature and 1000 rpm stirring speed).

Initially, the influence of oil phase compositions (*Eucalyptus* essential oil and grape seed oil) on micelles particle size of nanoemulsion system was preliminary examined. This study is aimed to determine the fixed value of oil composition, which those formulation resulted with smallest micelles particle size was chose to be further formulated in entire experiment. Oil composition was varied by combining different mass ratio of *Eucalyptus* essential oil and grape seed oil prior to emulsification. Otherwise, the composition and preparation conditions; oil and surfactant contents, stirring speed and temperature conditions were standardized at 3.0 wt. %, 1000 rpm, and 25 °C, respectively. Besides, the viscosity test of oil phase also conducted to determine the relation between oil phase viscosity and droplet size formation. Therefore, the influence of oil composition on micellar particle size and oil phase viscosity results are shown in Figs 4 and 5.

According to Fig. 4, smaller micellar nanoparticles of *Eucalyptus globulus* essential oil is succesfully formed (below 200 nm) in the nanoemulsion system. As the composition of *Eucalyptus* essential oil increases from 0.0 to 2.5 wt. %, the *Eucalyptus-*micelles droplets reduces significantly, however sudden increases up to 180 nm at single amount of *Eucalyptus* essential oil. Besides, smallest mean (42.85 nm) micelles particle size is reported to those formulation with 2.5 wt. % of *Eucalyptus* essential oil and 0.5 wt. % grape seed oil.

Despite of this finding, the difference of micelles particle size formation also reported to be influenced by oil phase viscosity properties. Based on Fig. 5, it was shown that the oil phase viscosity is increases as amount of *Eucalyptus* essential oil increases. Then, when comparing with particle size analysis, there is a significantly reduces of micelles droplet size around 140 to 50 nm, as oil phase viscosity increases at 0.5 to 2.5 wt. % of *Eucalyptus* essential oil composition. However, as the oil phase viscosity has further increment from 2.5 wt. % to 3.0 wt. % of *Eucalyptus* essential oil compostion, the micelles particle size increases to 180 nm. Thus, these results indicate that there is no simple correlation between oil phase viscosity and micelles particle size formation.

In foregoing, various investigations reported the conflict of relation between oil phase viscosity and micelles particle size formation. Several researchers proved that the more viscous oil involve through nanoemulsions system, smaller micelles particle size was formed, while another researchers reported as increased of particle size^38,39^.

The similar result with no directly correlation between oil phase viscosity and micelles particle size has been revealed by Saberi *et al*.^29^ and Davidov-Pardo^30^, however there are some explainations have been stated. Depicting to the researchers, a possible reason regarding the influence of oil phase viscosity to smaller micelles particle size formation in the nanoemulsion system is depending on the principal mechanism of micelles formation using spontaneous emulsification. Generally, several physicochemical characteristic of organic phase may contribute to the size of micelles formed in the system, in which the oil viscosity will influence the surfactant rate diffusion from the organic phase to a queous phase.

One might expect that lower viscosity of oil phase possess to fasten the surfactant diffused from organic to aqueous phase which reduce the oil-water interface boundary and inreases the interface mobility, resulted into more likely fine micelles droplets size will be formed^39^. The non-correlation between physicochemical characteristic of oil components and micelles particle size resulted is may be because of the properties of surfactant-oil-water (SOW) mixtures in the micellar phase region formation is more important than the properties of pure oil.

The physicochemical properties, component structure, and concentration of SOW mixture may be change over time and space after titration process of organic phase into aqueous phase^29^. Hence, the influence of physicochemical properties of oil phase to the micelles particle size formation through spontaneous emulsification method is still unclear and further basic research is recommended to be accessed in this aspect. Therefore, the 3.0 wt. % of oil phase composition containing 2.5 wt. % and 0.5 wt. % of *Eucalyptus* essential oil and grape seed oil, respectively was used in further analyses.

### Nanoemulsion system characterizations analyses

Manipulated surfactants type and concentration that been used through the research experimental was considered for micellar formulation of *Eucalyptus* essential oil characterizations through nanoemulsions system. The thermodynamic stability, viscosity, micelles particle size, pH measurement and morphology properties study of nanoemulsion system was evaluated depending on surfactant types and concentrations. Tween 40, 60 and 80 are among surfactant types used through the nanoemulsion system with different concentrations;(3.0, 6.0, 9.0, 12.0, 15.0, and 18.0) wt. %.

### Thermodynamic stability analysis

Thermodynamically stable of micellar formulation is dependent on the ability of nanoemulsion system to be in stable condition after being placed at different temperature conditions^40,41^. Through this research, thermodynamic stability analysis of the nanoemulsion system was accessed to determine the stable micellar formulation depending on manipulated surfactant types and concentrations in nanoemulsion system.

All nanoemulsion system have been placed at different thermodynamic stress conditions; centrifugation (CENT), heating-cooling cycle (HCC), and freeze-thaw temperature cycle (FT). The formulations considered stable were those passed all thermodynamic stress tests, and chosen to be used in the next characterizations analysis.

Depicting to Tables 2–4, *Eucalyptus-*micellar formulation with Tween 40 surfactant showed the highest thermodynamic stability of nanoemulsions system at all surfactant concentrations. However, those formulations developed by Tween 60 and Tween 80 surfactants have similar nanoemulsion stability pattern, whereas reported to be unstable at lower surfactant concentrations (3.0, 6.0, and 9.0 wt. %) but stable at higher surfactant concentrations (12.0, 15.0, and 18.0 wt. %).

**Table 2:** Optical properties of micellar formulation through nanoemulsion system by Tween 40 surfactant.

Oil Phase Composition (wt. %)	Surfactant Concentration (wt. %)	Thermodynamic Stability Phase Analysis			Optical Properties
		CENT	HCC	FT	
3.0	3.0	Pass	Pass	Pass	Stable
3.0	6.0	Pass	Pass	Pass	Stable
3.0	9.0	Pass	Pass	Pass	Stable
3.0	12.0	Pass	Pass	Pass	Stable
3.0	15.0	Pass	Pass	Pass	Stable
3.0	18.0	Pass	Pass	Pass	Stable

**Table 3:** Optical properties of micellar formulation through nanoemulsion system by Tween 60 surfactant.

Oil Phase Composition (wt. %)	Surfactant Concentration (wt. %)	Thermodynamic Stability Phase Analysis			Optical Properties
		CENT	HCC	FT	
3.0	3.0	Not Pass	Not Pass	Not Pass	Unstable
3.0	6.0	Not Pass	Not Pass	Not Pass	Unstable
3.0	9.0	Not Pass	Not Pass	Not Pass	Unstable
3.0	12.0	Pass	Pass	Pass	Stable
3.0	15.0	Pass	Pass	Pass	Stable
3.0	18.0	Pass	Pass	Pass	Stable

**Table 4:** Optical properties of micellar formulation through nanoemulsion system by Tween 80 surfactant.

Oil Phase Composition (wt. %)	Surfactant Concentration (wt. %)	Thermodynamic Stability Phase Analysis			Optical Properties
		CENT	HCC	FT	
3.0	3.0	Pass	Not Pass	Not Pass	Unstable
3.0	6.0	Pass	Not Pass	Not Pass	Unstable
3.0	9.0	Pass	Not Pass	Not Pass	Unstable
3.0	12.0	Pass	Pass	Pass	Stable
3.0	15.0	Pass	Pass	Pass	Stable
3.0	18.0	Pass	Pass	Pass	Stable

In spontaneous emulsification method, surfactant type is one of the most important parameter in formulating fine micelles particle size, and resulted into highly stable of nanoemulsions system. Tweens and Spans surfactants are the most suitable surfactant used in spontaneous emulsification method, regarded to their optimum surfactant geometry which efficient to possess spontaneous formation of micelles at oil and water interface^42^. Besides, stability of nanoemulsion system has been reported to be significantly correlate with smaller and uniform micelles particle size formed. This finding is supported by the Stoke’s Law that stated the gravitational separation of micelles droplet in the nanoemulsion system is directly proportional to the micelles particle radius. The formula is shown in Eq. 1.

1 $${{\rm{V}}}_{{\rm{STOKES}}}=\frac{2{\rm{gr}}2(\Delta p)}{9\eta }$$

where, V_stokes_: The rate velocity of micelles droplet moving upward

g: Gravity

r: Micelles particle radius

Δp: Dispersed phase

η: Shear viscosity of continous phase

Equation 1 shows direct correlation between stability of nanoemulsion system to the size of micelles droplets. Previous literatures have mentioned the smaller size of micelles promotes better stability of nanoemulsion system against coalescence or re-coalescence, gravitational separation, Ostwald ripening, and flocculation phenomenon^42,43^. Based on the current results (Tables 1–4) it is clearly shown that Tween 40 surfactant possessed a successful stability of nanoemulsion system, compared to Tween 60 and 80. Therefore, dependent on Eq. 1, this finding was attributable to smaller *Eucalyptus-*micelles particle size that may formed in Tween 40 nanoemulsion system. Through spontaneous emulsification method, different stability and micelles particle size formation developed by different surfactant type is due to two major reasons: surfactant hydrophilic lipophilic balance (HLB) and surfactant packing parameter (molecular geometry)^29^.

The highest stability of Tween 40 micellar formulation reported is depending on the surfactant hydrophilic lipophilic balance (HLB) factor. Among the surfactants, Tween 40 is reported to offer the highest HLB value (15.6), followed by Tween 80 (15), and Tween 60 (14.9). The HLB value of surfactant represents the hydrophobicity and hydrophilicity of non-ionic surfactant. Micelles formation through nanoemulsion system need higher HLB value to form a most stable solution, since the higher the HLB value, the higher the surfactant hydrophilicity and more likely to accumulate with aqueous environment and form smaller micelles particle size, and resulted into successsful thermodynamic stability^27^.

Hence, in this case, the highest HLB value of Tween 40, possess it to easily interact with aqueous solution and form the dispersion of micelles uniformly at the moment organic phase diffused into aqueous phase. This condition promoted the increases of adsorption rate between oil and water interface and resulted into decreases in the interfacial tension which possessed to the formation of fine *Eucalyptus-*micelles droplets formation^27^. Komaiko and McClements^43^ reported similar result whereas the nanoemulsion system with higher HLB value illustrated different micelles particle size formed at multiple surfactant types; Span 20 (59.16 µm), Tween 20 (1.46 µm), Tween 40 (0.10 µm), Tween 60 (0.23 µm), Tween 80 (0.12 µm), and Tween 85 (2.65 µm)^43^. Besides, another investigation formulated micelles of orange oil using various Tweens surfactant; Tween 20, 40, 60, 80, and 85 reported to form fine micelles particle size at those formulations developed by Tween 40, 60, and 80. This was attributable to higher HLB value of these surfactants compared to the other Tweens surfactant^35^. Another study by An *et al*.^44^ demonstrated that micellar nanoparticles formed by mixed surfactant solutions; Tween 80 and Span 20 was achieved smaller micelles particle size in nanoemulsion system containing 5 wt.% and 10 wt.% of MCT oil and surfactant, respectively after varying the mixed surfactant HLB value into 13.4.

Other than that, in the aspect of manipulated surfactant concentration used, all *Eucalyptus* micellar formulations are reported to become stable as increased of surfactant concentrations. This finding is significant with the other previous literatures, whereas the mean micelles particle diameter decreased with increasing surfactant concentration and formed stable nanoemulsion system through spontaneous emulsification technique^45,46^. Saberi *et al*.^29^ confirmed that the fine micelles droplets formed in nanoemulsion system was regarding to the higher reduction rate of oil-water interfacial tension as surfactant concentration increases, which facilitates to finer micelles formation. Similar result and explanation were stated by Sugumar *et al*.^45^ which developed micellar formulation of antimicrobial *Eucalyptus* essential oil in nanoemulsion system via spontaneous emulsification.

### Viscosity analysis

**Figure 6:**
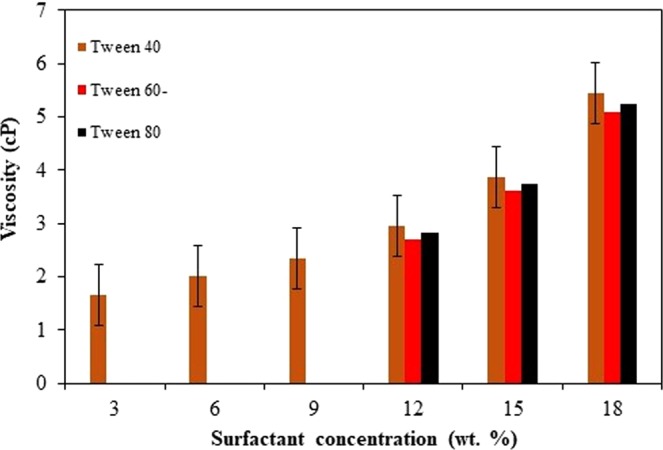
Nanoemulsions system viscosity dependent on manipulated surfactant concentrations (experimental conditions: 100 mL solution; 3.0 wt. % of oil composition, manipulated surfactant concentrations at 25 °C temperature and 1000 rpm stirring speed).

Viscosity is one of the important physicochemical characteristic for transdermal purpose of nanoemulsion system, to ensure the micellar formulation that being developed promotes pleasant skin feel after application. Through this research, those formulations with thermodynamically stable were chosen to undergo the viscosity analysis. The purpose of this analysis is to evaluate the effect of surfactant types and concentrations on viscosity of micellar formulation through nanoemulsion system. The viscosity analysis of nanoemulsions was accessed for all formulations of Tween 40 surfactant, and half of formulations of Tween 60 and 80 for their surfactant concentrations at 12.0 wt. %, 15.0 wt. %, and 18.0 wt. %, respectively. Depicting to Fig. 6, nanoemulsions viscosity increases which directly correlate to surfactants concentration increases at each surfactant type. Besides, the surfactant types influence nanoemulsions viscosity value, where Tween 40 formulation show the highest viscosity value (5.45 cP), followed by Tween 80 (5.25 cP) and Tween 60 (5.09 cP).

The higher viscosity formulation is may due to hydrogen bonding between surfactant hydrophilic segments with water molecules which possess the water molecules entrapped in the cross-linking portions of surfactant and causes formulation to become highly viscous^45^. More viscosity of micellar formulation is may attributable to more surfactant cross-linking portions as its concentration increased^46^. Additionally, this also can be the main reason of Tween 40 nanoemulsions resulted into the highest viscosity value, due to its highest HLB value compared to the another two surfactants. High HLB of surfactant contain more hydrophilic portions and possible to interact more with water molecules inside nanoemulsions system^47^.

A study demonstrated the increased of viscosity of citrus essential oil-micellar formulation (1.77 cP to 19.75 cP) through nanoemulsion system as surfactant concentration increased from 6.0 wt. % to 24.0 wt. %, which implemented the similar mechanism reason^35^. Besides, Sugumar *et al*.^45^ demonstrated a micellar formulation of *Eucalyptus* essential oil promoted higher viscosity, directly correlated to the increment of Tween 20 surfactant concentration through nanoemulsion system. However, all results were proved to be the lowest range of viscosity value, in which expected due to one of the excellent nanoemulsions characterization is lower viscosity and suitable for transdermal or topical administration purpose^48^.

### Micelles particle size and pH measurement analyses

A successful novel micellar formulation is depending to the fine mean micelles particle diameter formation and through spontaneous emulsification technique, therefore surfactant play the major role. Smaller diameter of micelles by successfully reduce interfacial tension between oil and aqueous phase can be facilitated by surfactant. Therefore, present evaluation showed the affection of surfactant types and concentrations on micelles particle size formation in nanoemulsion system. Besides, pH measurement of each micellar formulation is determined to ensure its suitability application for dermal and transdermal administration purposes. The result of is shown in Table 5.

**Table 5:** Micelles particle size, polydispersity index and pH values of *Eucalyptus* essential oil-micellar nanoparticle in nanoemulsion system.

Surfactant	Mass Fraction (Oil:Surfactant)	Micelles Average Particle Size (nm)	Polydispersity Index	pH Value
Tween 40	1:1	42.85 ± 0.2759	0.242 ± 0.0047	7.20
	1:2	19.92 ± 0.0686	0.222 ± 0.0038	6.95
	1:3	17.13 ± 0.035	0.305 ± 0.0056	6.57
	1:4	17.57 ± 0.0984	0.495 ± 0.0026	6.02
	1:5	13.68 ± 0.0759	0.377 ± 0.0026	5.47
	1:6	12.57 ± 0.0947	0.349 ± 0.0087	5.20
Tween 60	1:4	54.07 ± 0.3091	0.887 ± 0.0028	6.35
	1:5	68.5 ± 0.2160	0.935 ± 0.0104	6.01
	1:6	72.97 ± 0.0406	0.861 ± 0.2759	5.75
Tween 80	1:4	24.21 ± 0.1143	0.796 ± 0.0014	6.01
	1:5	23.51 ± 0.4852	0.738 ± 0.0086	5.42
	1:6	45.01 ± 21.144	0.548 ± 0.0786	5.13

According to Table 5, all surfactant types showed successful in resulting smaller *Eucalyptus-*micelles particle size (<200 nm) at each surfactant concentration. Through a micellar formulation containing Tween 40 at concentration of 18.0 wt. %, the finest micelles droplet was formed (d = 12.57 ± 0.0947 nm at PDI = 0.349 ± 0.0087), whereas the largest micelles particle size were formed in the system formulated with Tween 60 (d = 72.97 ± 0.0406 nm at PDI = 0.862 ± 0.2759) at concentration of 18.0 wt. %. Besides, formation of smaller micelles particle size was directly correlated with the increment of Tween 40 surfactant concentration, while resulted to be no correlation as Tween 60 and 80 concentrations increased.

Regarding to the surfactants type issue, these results indicate a strong correlation between the particle size and HLB value of surfactant. In some cases, HLB value of surfactant plays a major role in forming a stable and smallest micelles particle size in the nanoemulsion system. As discussed earlier, the hydrophobicity and hydrophilicity of non-ionic surfactants are depend on their HLB value system, where higher HLB value promoted more hydrophilicity of surfactant and vice versa^35^. Therefore, the higher HLB value of Tween 40 (15.6) compared to Tween 60 (14.9) possess it to be more hydrophilic and easier to interact with water molecules during the surfactant diffusion from organic to aqueous phase, and resulted into smaller micelles particle size are formed. As the surfactant hydrophilicity is higher, oil-water interface can be further reduced and facilitates fine micelles droplets formation.

Some previous studies support the current results that state the HLB value of surfactant encourage a potential of fine micelles particle size through nanoemulsion system. Chang and McClements^35^ demonstrated that the nanoemulsion containing orange oil and MCT formulated with intermediate high value of HLB surfactants; 15.6, 14.9, and 15.0 belonging to Tween 40, 60 and 80 resulted to the most stable nanoemulsion. Besides, a study conducted by Komaiko and McClements^43^ revealed smaller micelles particle size of different oils was affected by different types of surfactant used. Smallest emulsion diameter was reported to be formed in those formulation, formulated using Tween 40 and 80 which the most higher HLB value of surfactant compared to the other surfactants used.

Besides, different formation of micelles droplets size can be related to surfactant molecular geometry factor^43^. This finding is significant with the present study result which shows smaller micelles particle size formed by Tween 80 (HLB 15) nanoemulsion compared to Tween 60 (HLB 14.9) formulation, even though these surfactants have a similar polarity group. However, they are differ in their molecular geometry, where the hydrophobic segments of Tween 60 are saturated and therefore, fairly near. Meanwhile, the hydrophobic segments of Tween 80 are unsaturated and therefore resulted to more kinked which affect the surfactant packing on oil-water interface and may lead to tendency of fine micelles particle size formation via spontaneously produced when the organic phase is mixed with aqueous phase^43^. Previously reported by An *et al*.^44^, Saberi *et al*.^29^ and Chang *et al*.^35^, have been mentioned regarding Tween 80 surfactant have an optimum curvature and packing parameter that promote smaller micelles particle size in the nanoemulsion system.

Despite of this, there are two reasons have been mentioned regarding the impact of surfactant concentration to smaller micelles particle size formation in the nanoemulsion system. Firstly, it has been assigned to increased the surfactant adsorption rate onto oil-water interface for further decreased the interfacial tension, and facilitates to the fine micelles formation which significant to the increment of surfactant concentration. Second, more surfactant added to the nanoemulsion system, greater amount of surfactant diffused from organic phase to aqueous phase which lead to the formation of finer oil droplets^49,50^. However, different with Tween 60 and 80 nanoemulsion formulation where the average micelles particle size increases as the surfactant concentration increases. This is due to at certain above of surfactant amount added to the formulation, a highly viscous liquid crystalline can be formed which encourage to difficult in making the spontaneous breakup of the oil-water interface^29^. Besides, this result also may atrributable to lower HLB value of both Tween 60 and 80 surfactants, which indicate a lower hydrophilicity and possess to less interaction with aqueous environment, hence easily formed a liquid crystalline phase in the nanoemulsion system.

The enrichment of capsanthin with micellar nanoparticle in nanoemulsion system has been formulated by An *et al*.^44^ using spontaneous emulsification method, which developed smaller micelles droplets (38.5 nm) as surfactant concentration increased from 2.0 wt. % until 12.5 wt. %. Another study showed the direct correlation of surfactant concentration increased with vitamin D-micelles particle size, and exhibited an enhancement of vitamin D solubility and therapeutic potential^41^. Besides, the fish oil with physically stable and oxidize has been enriched with micellar nanoparticle in nanoemulsion system, whereas the increment of surfactant concentration was reported to be highly influenced for the formation of fine fish oil-micelles droplets^50^. Other than that, Sugumar *et al*.^45^ demonstrated smaller Eucalyptus-micelles particle size was reduced from 280.9 nm to 47.7 nm as Tween 20 surfactant concentration increased from 6 wt. % to 24 wt.% via spontaneous emulsification technique.

The investigation regarding bigger micelles droplets size resulted by surfactant concentration increases has been conducted by Saberi *et al*.^29^. This study has showed the vitamin E-micelles particle size increased at surfactant concentrations increased (60–130 nm) from 10.0 to 7.5 wt. %, which initially the micelles size reduced at concentrations below 10.0 wt. %. Besides, Gulotta *et al*.^51^ demonstrated an increment of omega three- micelles droplets as surfactant concentrations increased (5.0 to 15 wt. %) in nanoemulsion system via spontaneous emulsification method.

The polydispersity index (PDI) reported values did not follow the same trend as average micelles particle size, whereas a fluctuate PDI values are formed through overall formulations. However, all polydispersity index of nanoemulsion still demonstrate a good micelles droplets distribution since the PDI values did not exceed 1.0.

Previous study regarding thymol essential oil nanoemulsion also showed a similar trend of PDI values dependent on manipulated surfactant types and concentrations, which still formed a successful nanoemulsion formulation with ability to enhance the essential oil antibacterial pharmacological activity^46^. Besides, Saberi *et al*.^29^ and Guttoff *et al*.^41^ did not follow the same trend of polydispersity index, where the most narrow distribution of micelles occuring at smaller particle size which the PDI increased significantly with surfactant concentrations. Thus, a micellar formulation of Tween 40 surfactant at 9.0 wt. % is selected as optimum formulation since its ability to form successful thermodynamic stable of nanoemulsion system, suitable value of viscosity and pH for transdermal purposes, and smaller micelles particle size at lower surfactant concentration. This optimum formulation was chosen to be further analyzed for morphology analysis and *in vivo* analgesic study

### Morphology analysis

**Figure 7:**
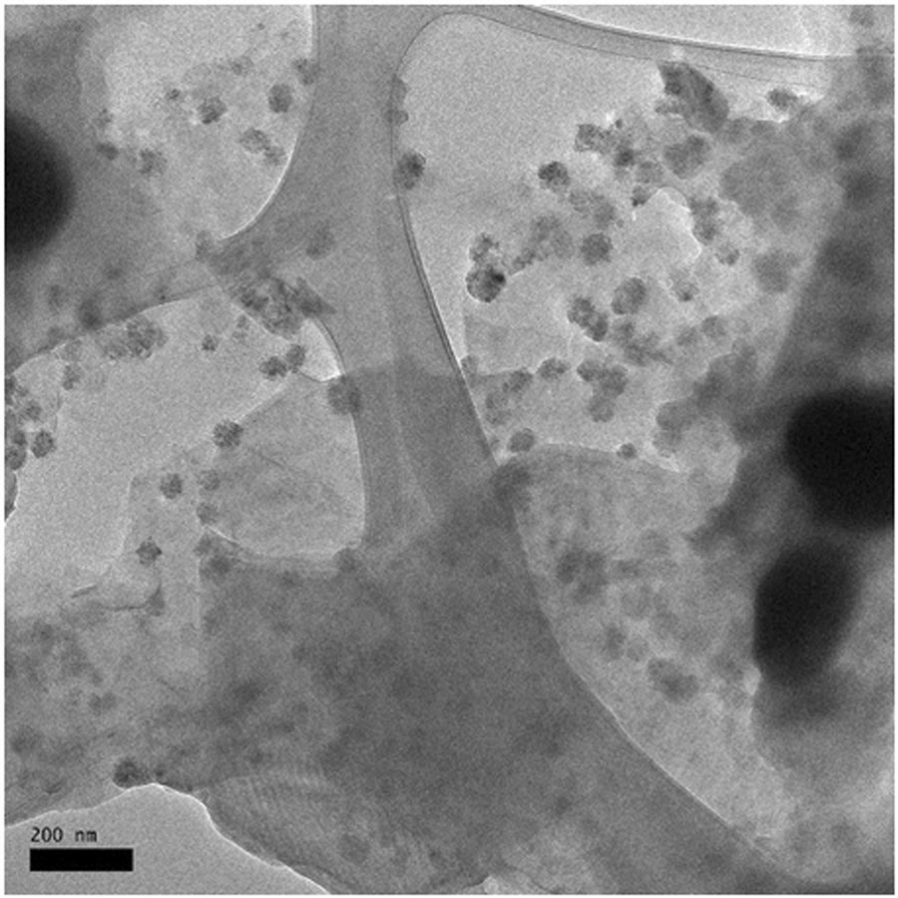
Morphology structure of *Eucalyptus* essential oil-micellar nanoparticle in nanoemulsion system.

Morphological structure of the optimum micellar formulation was determined by transmission electron microscopy (TEM). Figure 7 shows the TEM micrograph of optimized nanoemulsion formulation (Tween 40 at 9.0 wt. %) demonstrating the spherical shape of micellar droplets. The TEM result also confirmed the nanometric micelles diameter (« 20 nm) of formulated emulsion.

However, there was non-uniformity of micelles droplets dispersion within the *Eucalyptus-*micellar formulation in nanoemulsion system. This finding is significant with the larger polydispersity index (PDI) resulted (>0.2). A uniform or good homogeneity of micellar formulation can be achieved as the PDI value is below than 0.2^47^.

### *In Vivo* hot plate test in rats assay (analgesic activity evaluation)

**Figure 8:**
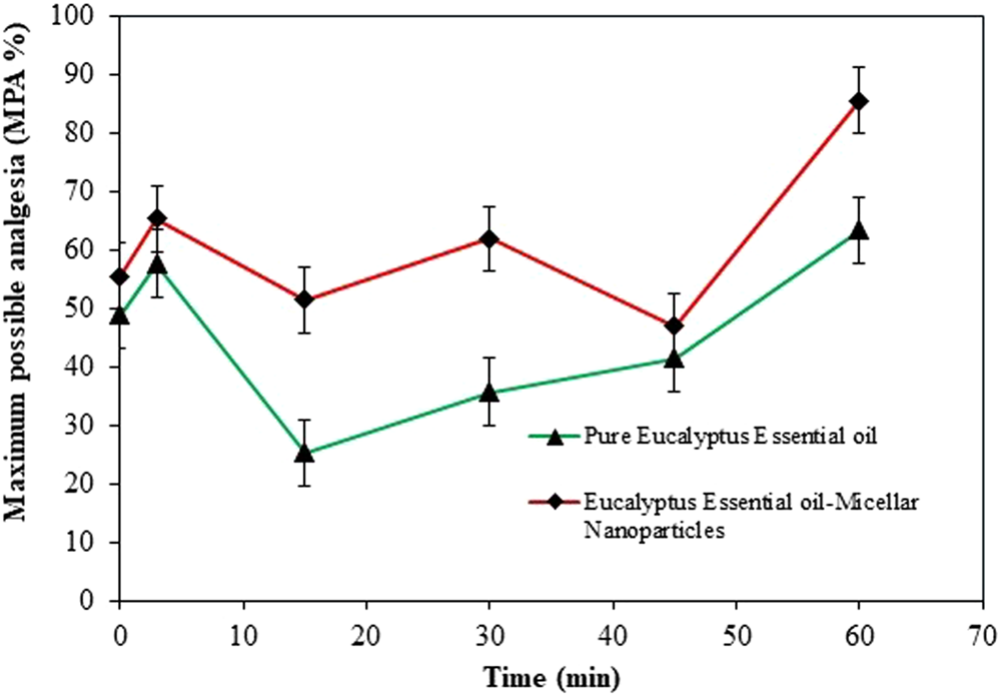
Maximum possible analgesia (MPA) (%) representing the comparison of analgesic activity between pure *Eucalyptus* essential oil and *Eucalyptus* essential oil-micellar nanoparticle transdermal administered into rats (experimental conditions: 100 mL solution; 3.0 wt. % of oil composition, 9. 0 wt. % of Tween 40 surfactant concentration, at 25 °C temperature and 1000 rpm stirring speed).

The efficacy of micellar nanoparticle in enhancing therapeutics of transdermal administration of *Eucalyptus* essential oil was evaluated by conducting *in vivo* analgesic activity assay. Table 6 shows the results of mean rats reaction time towards thermal stimulus pain after topically applied with different treatments, meanwhile Fig. 8 shows percentage of maximum possible analgesia (MPA) (%) between two groups of *Eucalyptus* essential oil at each time interval.

**Table 6:** Rats pain reaction time after topically applied with normal saline (10 mL/kg), pure *Eucalyptus* essential oil (500 mg/kg), and *Eucalyptus* essential oil-micellar nanoparticle (100 mg/kg).

Treatments	^a^Reaction time (s)					
	0	3	15	30	45	60
Normal saline (10 mL/kg)	22.5 ± 3.95	19.0 ± 2.95	24.0 ± 6.15	24.0 ± 3.61	17.3 ± 2.25	15.8 ± 3.02
Pure *Eucalyptus* essential oil (500 mg/kg)	33.5 ± 0.70	34.0 ± 2.25	29.3 ± 3.3	31.5 ± 2.18	28.8 ± 2.05	34.3 ± 3.05
*Eucalyptus* essential oil-micellar nanoparticle (100 mg/kg)	35.0 ± 4.10	36.0 ± 2.45	34.8 ± 3.45	37.0 ± 3.50	30.3 ± 3.55	40.8 ± 2.7

^a^Mean of rat’s pain reaction time.

According to Table 6, the transdermal administration of *Eucalyptus* essential oil-micellar nanoparticle (100 mg/kg) showed a significant prolonged time of rats responses towards the thermal pain, compared to normal saline (negative control) which reported to be no significant increase of reaction time within overall 60 min of treatment. Being compared with the positive control (pure *Eucalyptus* essential oil) at the dosage of 500 mg/kg, the potential of this pure aromatic oil to prolong the time responses of rats was slightly lower than micellar formulation of *Eucalyptus* essential oil.

The longest time responses reported by the formulated micellar of *Eucalyptus* essential oil was 40.75 s at 60 min after treatment administration, while it was 26 and 34.5 s reported for saline and pure *Eucalyptus* essential oil, respectively. Although the fabricated *Eucalyptus* essential oil with micellar nanoparticle appeared to induce higher analgesic potential in most cases, there was no consistency of the analgesic activity pattern of the micellar formulation. Therefore, in order to see further difference analgesic potential between pure *Eucalyptus* essential oil and micellar formulation of *Eucalyptus* essential oil, the maximum possible analgesia (MPA) percentage was calculated for these substances and the result is shown in Fig. 8.

Micellar nanoparticle that being enriched with the *Eucalyptus* essential oil may become the major reason of the better analgesic performance of *Eucalyptus*-micelles compared to its pure extract. As reported, micellar nanoparticle is a robust nanotechnological approach with high achievement of transdermal therapeutics, which enable to reduce essential oil volatility, toxicity, and enhance the solubility and bioavailability. Therefore, by enriching the *Eucalyptus* essential oil with micellar nanoparticle, its volatility rate may reduced while protecting the main constituent, 1,8-cineole during transdermally administered onto rats skin which promoted a prolong of rats pain analgesia.

Despite of this reason, the fine micelles particle size (17.15 nm) and higher stability of the optimized formulation may possess the enhancement of aromatic oil to be penetrated onto rats skin, and resulted into higher bioavailability and therapeutic analgesic potential achieved. Previously, Saberi *et al*.^29^ have been developed a vitamin E-micelles through nanoemulsion system via spontaneous emulsification method. They have reported that the bioavailability and therapeutic potential of encapsulated bioactive constituent can be enhanced as the micelles droplet size in emulsion decreases, although this is likely to highly system dependent.

Overall, the successful analgesic potential of the micellar formulation containing *Eucalyptus* essential oil in the animal model testing, suggest its inhibitory effects on both central and peripheral analgesic activities. Furthermore, previously investigation on transdermal analgesic activity of doxepin-micellar nanoparticle in the nanoemulsion system revealed that the nanoemulsion containing smaller micelles of doxepin showed a successful analgesic potential compared to doxepin control group by using the same animal model method^52^.

Additionally, another study reported the significant difference of transdermal ketoprofen-nanoemulsion as analgesic agent was better than control group (marketed ketoprofen product, Fastum® gel) through different animal testing (carrageenan-induced hyperalgesia pain threshold test)^53^_._

## Discussion

Eucalyptol or 1,8-cineole is a phytochemical constituent that mainly found as main component in *Eucalyptus globulus* essential oil, acting as natural analgesic agent^6^. Despite its pharmacological benefit, efficacy and bioavailability of *Eucalyptus globulus* essential oil are affected by its lipophilic, highly volatility and easily decomposed characteristics. Encapsulation is a better approach to enhance the essential oil therapeutic potential without altering its biological properties^19^. Micellar nanoparticles is one of latest nanotechnology approach to be enriched with lipophilic component so that the bioavailability can be enhanced^22^. Therefore, the present study commenced to formulate micellar nanoparticles of *Eucalyptus globulus* essential oil in nanoemulsion system via spontaneous emulsification technique. Nanoemulsion can form micellar nanoparticles by low and high energy techniques through dispersion of one liquid to another immiscible liquid phase. Among the emulsification techniques, spontaneous emulsification method consider as simple, cost-effective and low-energy approach that suitable for essential oil encapsulation due to essential oil’s labile properties^29^. Through the present study, 1,8-cineole was found to be the main constituent contained inside the *Eucalyptus globulus* essential oil with 75.96%. Spontaneous emulsification technique was successfully developed a transparent formulation of nanoemulsion system, contained dispersion of *Eucalyptus globulus* essential oil- loaded micellar nanoparticle through aqueous solution. Preliminary experiment was conducted to determine the fixed value of oil composition, which those formulation resulted with smallest micelles particle size was chose to be further formulated in entire experiment.

The characterizations of nanoemulsion system such as thermodynamic stability, viscosity, micelles particle size, pH value and morphological structure were affected by manipulated surfactants type and concentration. In terms of surfactants type factor, different hydophilic-lipophilic balance (HLB) value and molecular geometry of each surfactant (Tween 40, 60 and 80) used play as major role. Those *Eucalyptus-*micellar formulation with higher HLB value (Tween 40) of surfactant promoted higher stability, smaller micelles particle size and spherical morphological structure formed throughout the nanoemulsion system. This is attributable to higher HLB of surfactant that possessed to be easily interact with water molecules during the surfactant diffusion from organic to aqueous phase, and resulted into smaller micelles particle size are formed^29^. As the surfactant hydrophilicity is higher, oil-water interface can be further reduced and facilitates fine micelles droplets formation^43^. Hence, the smaller the micelles particle size formed, the higher stability of nanoemulsion system. Besides, it also has been reported that the molecular geometry of surfactant contributed to form smaller micelles particle size, especially for surfactants with similar polarity. Formulation with Tween 80 surfactant reported to form smaller dispersions of *Eucalyptus-*micelles in the nanoemulsion system compared to Tween 40 formulation, eventhough both of them have almost similar HLB value. However, since the molecular geometry of Tween 80 surfactant containing unsaturated hydrocarbon segments and more kinked, it can affect the surfactant packing on oil-water interface and may lead to tendency of fine micelles particle size formation via spontaneously produced when the organic phase is mixed with aqueous phase^27^.

In the aspect of surfactants concentration, it was reported that an increased of surfactant concentration was lead to higher stability, more viscous and smaller micelles particle size successfully developed in the nanoemulsion system. Firstly, it has been assigned to increased the surfactant adsorption rate onto oil-water interface for further decreased the interfacial tension, and facilitates to the fine micelles formation which significant to the increment of surfactant concentration^29^. Second, more surfactant added to the nanoemulsion system, greater amount of surfactant diffused from organic phase to aqueous phase which lead to the formation of finer oil droplets^29^. However in the some aspect, an increased of surfactant concentration can possessed to increased with micelles particle size. This was attributable to at certain above of surfactant amount added to the formulation, a highly viscous liquid crystalline can be formed which encourage to difficult in making the spontaneous breakup of the oil-water interface and resulted into bigger micelles particle size formation^29^. Hence, the role of surfactants type and concentration on nanoemulsion system characterizations has been well mentioned in previous studies^29,30,35,43,44,46^.

Besides, the nanoemulsion system was reported to be more viscous as the formulated micellar nanoparticles of *Eucalyptus* essential oil inreased with surfactant concentration. Those formulation with Tween 40 surfactant was reported to be higher in viscosity compared to Tween 60 and 80 surfactants. This is attributable to more surfactant cross-linking portions as its concentration increased and causes the formulation became more viscous. Additionally, HLB value of surfactant used was also affected the formulation viscosity. As higher the HLB of surfactant, it contain more hydrophilic portions and possible to interact more with water molecules inside nanoemulsions system, created more hydrogen bonding and possessed higher viscosity of micellar formulation^46^. However, all results were proved to be the lowest range of viscosity value, in which expected due to one of the excellent nanoemulsions characterization is lower viscosity and suitable for transdermal or topical administration purpose^49^.

Therefore, it should be noted that these formulated *Eucalyptus* essential oil-micellar nanoparticle in the nanoemulsion system are able to form small mean micelles particle size (<200 nm) at lower concentration. These results indicate that the micellar nanoparticle of *Eucalyptus* essential oil in the nanoemulsion system can be successfully formed by spontaneous emulsification method even at relatively lower concentration, which may be important for some commercial application. Other than that, the pH value resulted by all formulations are suitable to be used for dermal or transdermal administration due to their value ranges 6–7 according to the standard.

Hence, these nanoemulsion characterizations evaluations show the significant impacts of surfactant types and concentrations to the thermodynamic stability, viscosity, and micelles particle size formation. As discussed earlier, an optimized formulation with stable thermodynamic stability, suitable viscosity and pH value, and smaller average micelles particle size results will be chosen to be further analysed for morphology study and animal testing. Thus, a Eucalyptus-micellar formulation of Tween 40 surfactant at 9.0 wt. % is selected as optimized formulation since its ability to form successful thermodynamic stable of nanoemulsion system, suitable value of viscosity and pH for transdermal purposes, and smaller micelles particle size at lower surfactant concentration. This optimum formulation was chosen to be further analyzed for its analgesic activity properties.

Hot plate test in rats is one of *in vivo* analgesic study that assessed by measuring the rats’ pain responses towards thermal stimulus after being put on top of the hot plate. The Maximum Possible Analgesia (MPA) result showed a fluctuate pattern, whereas the analgesic effect of *Eucalyptus* essential oil pure extract and its micellar formulation started within 3 min and decreased towards 15 min after the treatments. Then, there was continuous increment of analgesic activity of pure *Eucalyptus* essential oil beginning at 30 min (35.71%) towards 60 min (63.36%). Meanwhile for *Eucalyptus* essential oil-micellar nanoparticle formulation, its possible analgesic effect was increased after 15 min (51.43%) of treatment to 30 min (61.90%), however decreased at 45 min (46.93%) and finally gradually increased at 60 min (85.62%) as the highest analgesic percentage. Besides, it was demonstrated that the analgesic potential of transdermal administration of formulated *Eucalyptus* essential oil-micellar nanoparticle reported to be higher compared to the pure *Eucalyptus* essential oil at each time points dependent on the MPA result.

Analgesics are substances that act on central nervous or peripheral system to possess a relieve pain action without any altering consciousness. The analgesic acting on central nervous system is due to increment in the threshold for pain and besides changing the physiological pain response. Meanwhile, the peripheral system acting analgesic by inhibiting the rate of impulses at pain chemoreceptor site^54,55^. The hot plate test conducted in this study involves a higher brain function of rats which resulted into supraspinally organized responses such as jumping, licking paw and extremely grooming.

Depicting to the finding, it was found that both micellar formulation of *Eucalyptus* essential oil and its pure extract demonstrated a good analgesic activity, however the micellar was reported to possess a better performance eventhough the dosage of pure extract (500 mg/kg) was higher than *Eucalyptus*-micelles (100 mg/kg). This may attributable to the 1,8-cineole content, a main biological constituent of *Eucalyptus* essential oil that still existed or only slightly evaporated after enriched with micellar nanoparticle, in which the 1,8-cineole is reported as a main analgesic pharmacological activity constituent of the aromatic oil^6^.

Overall, the successful analgesic potential of the micellar formulation containing *Eucalyptus* essential oil in the animal model testing, suggest its inhibitory effects on both central and peripheral analgesic activities. Furthermore, previously investigations showed an enhancement of transdermal analgesic of doxepin and ketoprofen after being fabricated with micellar nanoparticles on transdermal analgesic activity^52,53^.

Thus, these findings indicate that the micellar nanoparticle formation through nanoemulsion system can be suggested as innovated nanocarriers for pharmacological natural analgesic constituents in the treatment of alleviating pain.

## Conclusions

The enrichment of Eucalyptus globulus essential oil with micellar nanoparticles in nanoemulsion system with successful physicochemical properties of higher stability, smaller particle size, spherical morphological structure and suitable pH and viscosity values was successfully developed using spontaneous emulsification technique. These characteristics possessed a good potential of micellar nanoparticles as transdermal nanocarrier in efficiently demonstrated better analgesic activity of *Eucalyptus globulus* essential oil by prolonged the pain responses of rats towards thermal stimulus after being put on top of hot plate. Hence, these considerations can be used for the rational design of nanoemulsion system in forming the micellar nanoparticle-associated essential oil as based delivery system on the desired function of the analgesics in the cosmetics and pharmaceutical industries.

## Materials and Methods

### Materials

The oil components; *Eucalyptus globulus* essential oil and grape seed oil were purchased from YKL Personal Care Sdn Bhd, while surfactants; Tween 40, 60 and 80 were purchased from VNK Supply Sdn Bhd. Deionized water was supplied by Centre of Lipid Engineering and Applied Research laboratory, Universiti Teknologi Malaysia, Skudai. Normal saline used in animal testing was supplied by physiology laboratory, Universiti Putra Malaysia, Serdang.

### Gas chromatography-mass spectrometry (GC-MS) analysis of eucalyptus globulus essential oil

*Eucalyptus globulus* essential oil constituents were analysed by using gas chromatography mass spectrometry on a Hewlett-Packard 5971 instrument with a dimethylpolysiloxane DB-1 coated fused silica capillary column (30 m × 0.25 mm) and He as the carrier gas (1 mL/min). The injector temperature was 250 °C and the detector temperature was 200 °C. The column temperature program was 35–180 °C at 4 °C/min, then 180–250 °C at 10 °C/min. For mass spectrometry, the electron impact was 70 eV. This procedure was conducted depicted to previous investigation^32^.

### Formulation of eucalyptus essential oil-enriched micellar nanoparticle in nanoemulsion system by spontaneous emulsification technique

A greener micelles formation technique was applied by using spontaneous emulsification technique, and the procedure was same as previously reported with slightly modifications^45^. Through this, the organic phase contained *Eucalyptus globulus* essential oil (active constituents), grape seed oil (carrier oil) and surfactant wisely titrated into deionized water (aqueous phase) while magnetically stirred. The formulation was continuously stirred for 30 min. All formulated emulsions were manipulated for their different surfactant types (Tween 40, 60 and 80) and concentrations (3.0, 6.0, 9.0, 12.0, 15.0, and 18.0 wt. %) with constant oil composition (3.0 wt. %). The Tween 40, 60 and 80 surfactants have been used due to their HLB value that suitable for formation of micelles in O/W nanoemulsion, besides their non-toxic and irritant properties that preferable for transdermal application. Unless otherwise stated, the preparation conditions through emulsification process were fixed at 1000 rpm and 25 °C of stirring speed and temperature, respectively. The surfactants type and concentrations range used was dependent on previous studies which implemented similar parameters that reported to successfully developed transparent and smaller micelles particle size formation^36,46^.

### Preliminary experiment

Preliminary experiment was involved the manipulated composition of each mass fraction of oil components; *Eucalyptus globulus* essential oil and grape seed oil at the total oil composition of 3.0 wt. %. This total oil composition (3.0 wt. %) was dependent on the highest amount of *Eucalyptus globulus* essential oil recommended to be diluted in 100 L of warm water as bath additive and topical application for analgesic purposes, according to European Medicines Agency, 2013. This analysis was aimed to determine the fixed composition of each oil component to be used for entire research experiment. Micelles particle size was considered as the dependent parameter to choose those formulation with smallest particle size for further analysis. Unless otherwise stated, several process composition and conditions were fixed at total oil composition (3.0 wt. %), surfactant type (Tween 40); surfactant concentration (3.0 wt. %), and preparation conditions of temperature and stirring speed at 25 °C and 1000 rpm, respectively.

### Nanoemulsion characterization

This characterization step was aimed to choose an optimize *Eucalyptus* oil-enriched micellar nanoparticle formulation in nanoemulsion system, dependent on the effects of surfactants type and concentration to the thermodynamic stability study, viscosity, micelles particle size, pH measurement and morphology of emulsions. Further explanation regarding procedures involved for each characterization are discussed.

### Thermodynamic stability study

Thermodynamic stability for the micellar formulation of *Eucalyptus globulus* essential oil through nanoemulsion system was accessed by using thermal and stress tests. Initially, formulated emulsions were centrifuged at 3500 rpm for 30 min and was observed for phase separation if any. The procedure was followed by heating-cooling cycle method, where the sample was kept at 40 °C and 4 °C alternatively for two days. Then, freeze-thaw cycle was performed by keeping the emulsion at −21 °C and 25 °C for two days, alternatively at each temperature. Those formulations showing no phase separation, flocculation, coalescence, and cracking formulation were selected for further characterizations analyses^46^.

### Viscosity analysis

The viscosity of *Eucalyptus*-micellar formulations was measured by using Brookfield Viscometer without dilution. Viscosity measurements were recorded for triplicates^46^.

### Micelles particle size analysis

Micelles particle size distributions of nanoemulsions system were measured using a dynamic light scattering instrument (Zetasizer Nano ZS, Malvern Instrument). This instrument determines the particle size from intensity-time fluctuations of a laser beam (633 nm) scattered from a sample at an angle 173°. The mean micelles particle diameter (Z average) and polydispersity index (PDI) were calculated from the particle size distribution. The PDI value provides a measure of the narrowness of the particle size distribution, with value ≤0.1 indicating a very narrow distribution. All measurements were conducted after overnight storage of samples at ambient temperature^29^.

### pH measurement analysis

pH value of the emulsion was measured by a pH meter (model 361, Systronic). The experiment was done in triplicates. This was conducted to ensure the suitable range of pH value of formulated emulsions in dermal or transdermal application. Therefore, an optimized formulation was chosen to be further analysed for its morphology and efficacy testing. This was depicted to those formulation at most stable condition, smaller micelles particle size with suitable viscosity and pH values for transdermal administration characterizations, which formulated at acceptable surfactant concentration (not too high).

### Morphology analysis

Transmission Electron Microscopy (TEM) was carried out to visualize the shape and morphology of *Eucalyptus* essential oil-micelles dispersion through nanoemulsion system. To conduct the TEM observations, one drop of emulsion was negatively stained with phosphotungstic acid and was placed on top of copper grid. TEM micrographs were acquired by using a transmission electron microscope (Tecnai-10, Philips) with a tungsten source and operating at 200 kV^48^.

### Animal laboratory testing

Four week old male Sprague Dawley rats (n = 18) were purchased from the Animal Research Centre, Faculty of Veterinary Medicine, Universiti Putra Malaysia. The rats were housed under standard laboratory conditions at room temperature with relative humidity 60–70% and free access to standard rat diet and water *ad libitum*. The rats were randomly divided into three groups with each contained of six rats, and these groups were dependents on three treatments that being topically applied on top of fore and hind limb of rats. The treatments include; (i) normal saline (10 mL/kg) as negative control; (ii) pure *Eucalyptus* essential oil (500 mg/kg) as positive control and (iii) *Eucalyptus* essential oil-micellar nanoparticle (100 mg/kg). The experiments were performed in accordance with guidelines outlined and approved by Institutional Animal Care and Use Committee (Ref: UPM/IACUC/AUP-R013/2018) of Universiti Putra Malaysia, Malaysia.

### *In Vivo* hot plate test in rats assay (analgesic activity evaluation)

The animal experimental laboratory known as hot plate test has been accessed to employ an analgesic performance of rats using pain state model by thermal application^55^. Several treatments included topically applied of; (i) normal saline (10 mL/kg) as negative control; (ii) pure *Eucalyptus* essential oil (500 mg/kg) as positive control and (iii) *Eucalyptus* essential oil-micellar nanoparticle (100 mg/kg) on top of fore and hind limb of rats. Then, the rats were placed on top of hot plate at constant temperature of 55 °C. The reaction time (s) or latency period was determined as the time taken for the rats to response to the thermal pain by licking their paw, jumping or extremely grooming. The reaction time was recorded for 0.0, 3.0, 15.0, 30.0, 45.0, and 60.0 min of time intervals after treatments administration. Maximum reaction time was fixed at 45 s to prevent any injury to the tissues of rats paw. If the reading exceeds 45 s, it would be considered as maximum analgesia. The maximum possible analgesia (MPA) was calculated in Eq. 2.

2 $${\rm{MPA}}=\frac{Reaction\,time\,for\,treatment-reaction\,time\,for\,saline}{45\,\sec \,-reaction\,time\,for\,saline}\times 100$$
